# A Review of the Diagnostic Approaches for the Detection of Antimicrobial Resistance, Including the Role of Biosensors in Detecting Carbapenem Resistance Genes

**DOI:** 10.3390/genes16070794

**Published:** 2025-06-30

**Authors:** Kaily Kao, Evangelyn C. Alocilja

**Affiliations:** 1 Department of Biosystems and Agricultural Engineering, Michigan State University, East Lansing, MI 48824, USA; kaokaily@msu.edu; 2 Global Alliance for Rapid Diagnostics (GARD), Michigan State University, East Lansing, MI 48824, USA

**Keywords:** carbapenem resistance genes, antimicrobial resistance, diagnostics, biosensor, nanoparticles, One Health

## Abstract

Antimicrobial resistance (AMR) is a rapidly growing global concern resulting from the overuse of antibiotics in both agricultural and clinical settings, the lack of surveillance for resistant bacteria, and the low quality of some available antimicrobial agents. Resistant pathogens are no longer susceptible to common clinical antimicrobials, which decreases the effectiveness of medicines used to treat infections caused by these organisms. Carbapenems are an important class of antibiotics due to their broad-spectrum effectiveness in treating infections caused by Gram-positive and Gram-negative organisms. Carbapenem-resistant bacteria have been found not only in healthcare but also in the environment and food supply chain, where they have the potential to spread to pathogens and infect humans and animals. Current methods of detecting AMR genes are expensive and time-consuming. While these methods, like polymerase chain reactions or whole-genome sequencing, are considered the “gold standard” for diagnostics, the development of inexpensive, rapid diagnostic assays is necessary for effective AMR detection and management. Biosensors have shown potential for success in diagnostic testing due to their ease of use, inexpensive materials, rapid results, and portable nature. Biosensors can be combined with nanomaterials to produce sensitive and easily interpretable results. This review presents an overview of carbapenem resistance, current and emerging detection methods of antimicrobial resistance, and the application of biosensors for rapid diagnostic testing for bacterial resistance.

## 1. Introduction

Antimicrobial resistance (AMR) has developed as one of the most urgent threats to worldwide public health [1]. In 2019, the Centers for Disease Control and Prevention (CDC) estimated that in the United States, AMR affects more than two million people every year, with at least 35,000 deaths as a direct result [2]. Acquired AMR occurs when organisms, like viruses, parasites, fungi, and bacteria, adapt to grow in the presence of medicines that once impacted them [1]. The resistance to antimicrobials reduces the effectiveness of these treatments, leading to increased morbidity and mortality in patients with infections [3]. With the increase in antimicrobial resistance, the magnitude and impacts of AMR on global healthcare costs and outcomes have escalated [4]. There are several programs designed to improve AMR surveillance and develop proactive strategies to minimize its threats [4]. These proactive strategies include next-generation sequencing (NGS) technology, bioinformatics tools, and public databases keeping track of AMR [4].

There are several human contributors to AMR, including a lack of monitoring of resistant pathogens, low-quality of some antimicrobial agents, and misuse and ease of availability of antibiotics [5]. Many developing countries do not have the necessary infrastructure for antibiotic quality assurance, which results in the substandard antibiotic formulations [5]. It was shown that using subpar and expired antibiotics increased resistance rates compared to standard antibiotics by two to six times [5]. The growth of the human population and urbanization provide an increased opportunity for the spread of infectious diseases [6]. This ultimately leads to an increased use of antibiotics and an increased risk of overprescription by physicians [6]. Antibiotics are used in the clinical setting to treat infections and are essential for life-saving surgeries and medical procedures [7]. In 2019, the World Health Organization (WHO) categorized antibiotics by clinical importance, ranging from important to critically important [8]. Some of the critically important antimicrobial classes include aminoglycosides, carbapenems (beta-lactams), cephalosporins, glycopeptides, macrolides, penicillins, and quinolones [8]. Beta-lactams are the most frequently used antibiotic class in healthcare because of the low cost of production and their effectiveness [9].

In addition to clinical misuse, there is an overuse of antibiotics in agricultural applications [10]. Antibiotics provide an increase in crop productivity, the improvement of animal and livestock health, and promote growth in animals through feed, and this overuse has caused a significant increase in resistant microbes [10]. Prioritizing research on antimicrobials that are critically important to medical applications can be a risk management tool to aid in controlling antibiotics that should be used in food-producing animals [8].

The use of medically relevant antibiotics for growth promotion has been banned in the U.S. since 2017; however, non-medically relevant antibiotics can still be used for growth promotion [11]. Antibiotics provide benefits for animal health, are crucial for treating disease, and improve animal feed efficiency [12]. However, the high use of antibiotics in the agricultural industry has caused an increase in resistant organisms that can be transferred to humans [12]. The classes of antibiotics that are used in agriculture for disease treatment and prevention on a global level include tetracyclines, aminoglycosides, beta-lactams, lincosamides, macrolides, pleuromutilins, and sulfonamides [13]. Many of these antibiotics are medically relevant, meaning they are used for human medicine [14]. As the resistance to these antibiotics increases, there is growing concern that resistant bacteria may spread to humans either through direct contact with animals or indirectly through the handling of contaminated animal products [14]. In the U.S., there are specified antibiotics allowed in the agricultural industry, and the beta-lactam class of antibiotics has gained specific regulations due to the increasing resistance of medically important drugs in the subclass of carbapenems [15]. Only three beta-lactam antibiotics, including penicillin preparations, cephapirin, and ceftiofur, are approved for treating infections in livestock and farm animals in the U.S. [15]. In 2020, the FDA reported that the most purchased medically relevant antibiotics in the U.S. agricultural industry were tetracyclines, penicillins, macrolides, sulfonamides, and aminoglycosides [16]. Although some medically relevant antibiotics are no longer used in the agricultural industry in the U.S., resistant bacteria can be transported across international borders through trade and travel from countries without antibiotic use restrictions [14]. Trading animal products with countries without antibiotic restrictions could introduce AMR bacteria that developed a resistance to antibiotics restricted in the U.S. but not in the source country. It has also been shown that bacterial antibiotic resistance can persist and spread in livestock environments years after stopping antibiotic use [17]. Monitoring AMR is important not only in clinical settings but in the food supply chain and agricultural industry as well [18]. Several programs exist for AMR monitoring and are discussed in later sections.

There are also environmental contributors to AMR, and it is believed that AMR genes began with environmental bacteria that produce antibiotics when they compete for nutrients [19]. It was shown that antibiotic-resistant bacteria exist in pristine environments like untouched Arctic settings, which indicates that resistance mechanisms existed in microbial ecosystems before antibiotics were around [20]. Environments with large numbers of bacteria that could contain AMR include hospital wastewater, aquaculture, agricultural wastewater, and manure runoff from agricultural settings [19]. If surface and runoff water come into contact with municipal water, bacteria with AMR can spread through drinking water and food products if resistant bacteria are not removed before consumption [21].

**Figure 1:**
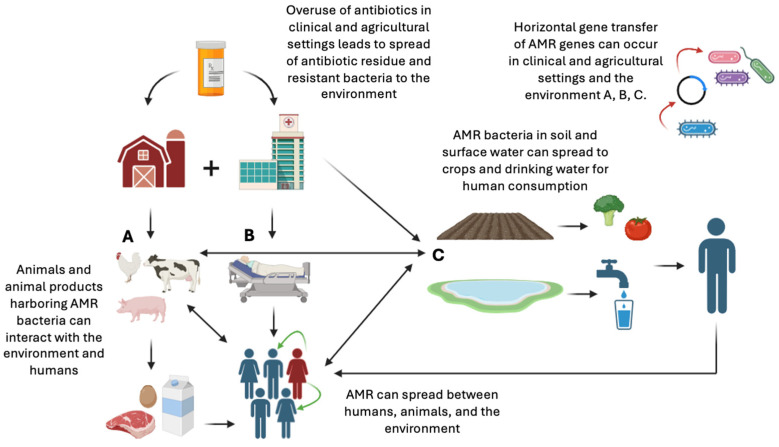
Potential pathways of how antimicrobials and resistant organisms travel through the environment and cause the spread of resistance genes and resistant infections (original image created with https://www.biorender.com, URL accessed on 12 June 2025).

Figure 1 illustrates a potential pathway of the spread of antimicrobial resistance.

The aim of this review is to provide an overview of carbapenem resistance, current and emerging detection methods of antimicrobial resistance, and the application of biosensors for rapid diagnostic testing for bacterial resistance.

## 2. Carbapenem Resistance

Beta-lactam antibiotics are the most frequently used class of antibiotics for human infection treatments, account for 65% of prescriptions for injectable antibiotics in the U.S., and are generally well tolerated with a low toxicity [22]. Subclasses of beta-lactam antibiotics include penicillins, monobactams, cephalosporins, and carbapenems [22]. Carbapenems are broad-spectrum treatments for Gram-positive and Gram-negative anaerobic bacteria [23]. The most commonly used carbapenems in clinical practice are meropenem, imipenem, doripenem, and ertapenem, which are frequently used for treating multi-drug-resistant infections [24]. Carbapenem resistance is a significant concern, as these antibiotics are considered a last resort treatment for severe infections [25].

### 2.1. Beta-Lactam and Carbapenem Resistance Genes in Bacteria

Acquired resistance is caused by gene mutations or infections caused by bacteria containing resistance plasmids [26]. Beta-lactam antibiotics are primarily used for treating bacterial infections, but the increased resistance has caused concern [27]. Beta-lactam antibiotics contain a four-member beta-lactam ring and target the cell wall of bacteria by preventing peptidoglycan cross-linking in cell wall synthesis [22,28]. Some of the most common resistance enzymes produced in bacteria are beta-lactamases, which inactivate beta-lactamase antibiotics [29]. Beta-lactamase-producing organisms are likely to display a resistance to beta-lactam and other classes of antibiotics [30]. Out of all beta-lactams, carbapenems are the most effective, broad-spectrum antibiotic for Gram-positive and Gram-negative bacteria [31]. Carbapenems have an increased stability against inactivating enzymes compared to beta-lactams in general [32]. Carbapenem-resistant (CR) bacteria produce enzymes that can inactivate both carbapenems and beta-lactams, and these enzymes are called carbapenemases [32]. These enzymes are encoded by genes, which can be present on mobile elements of bacterial genomes [33]. Mobile elements, like plasmids, promote intercellular exchanges of resistance genes, which is a major contributor of the spread of antibiotic resistance in bacteria [34]. While resistance genes are commonly present on plasmids, they can also be located on bacterial chromosomes [35]. It was shown that chromosome-located *bla*_NDM_ was associated with a lower carbapenem resistance and carbapenemase activity than plasmid-located *bla*_NDM_; however, both cases should be monitored [35]. Beta-lactam-resistant genes are separated into classes A, B, and D [36]. Class A (serine penicillinases) includes KPC-producing genes; class B (metallo-beta-lactamases) includes NDM-, IMP-, and VIM-producing genes; and class D (oxacillinases) includes OXA-producing genes [30,36]. Most carbapenem-resistant genes, including *bla*_KPC_, *bla*_NDM-1_, and *bla*_OXA-1_, are located on plasmids [37,38,39,40]. Of all carbapenemases, the KPC family is the most prevalent and found mostly on *Klebsiella pneumoniae* (*K. pneumoniae*) plasmids [41]. Based on a global surveillance study in Gram-negative pathogens from 2012 to 2014, KPC-2 and KPC-3 were the most common of the KPC carbapenemases [42].

Of the class B carbapenemases, NDM is the most common in *Enterobacteriaceae* [43]. Of the NDM carbapenemases, NDM-1 is the most common [44,45]. In the U.S., KPC is the most common carbapenemase produced by *Enterobacteriaceae*, overall, but outbreaks of NDM- and OXA-producing *Enterobacteriaceae* have also been reported [46]. Depending on the carbapenemase present, different combinations of antibiotics can be used to treat infections from CR bacteria [47].

### 2.2. CR in the Environment and Food Supply Chains

CR organisms have been found in the environment and food sources like seafood and aquaculture, food-producing animals, wildlife, and companion animals [48]. In the U.S., studies screening for carbapenem resistance in livestock found carbapenem resistance genes (CRGs) in cattle feces and pen soil [49,50]. Also in the U.S., CRGs were isolated from companion animals in a study using canine and feline clinical isolates [51]. A study on coastal marine environments, in Australia, found CR Gram-negative bacteria in several ocean waters, land runoff waters, and stormwater samples [52]. A study on shrimp from Vietnam showed that the shrimp were contaminated with *Enterobacter cloacae* (*E. cloacae*) resistant to carbapenems, ampicillin, and cefoxitin [53]. In Poland, multiple CRGs were found in community and river water, during a prevalence analysis study for carbapenem resistance genes in *Acinetobacter* spp. [54]. In Italy, on swine farms using medicated feed, CR *Aeromonas veronii* (*A. veronii*) was found in swine liver samples during a study analyzing the feces, feed, and animal-food derived products [55]. In Germany, a foodborne outbreak of CR *Citrobacter fruendii* (*C. fruendii*) occurred and was linked to sliced vegetables [56]. Antibiotic resistant genes in the environment and the food chain can be transferred to human pathogens and lead to increased human antibiotic resistance [57,58]. Food products have been identified as a potential source of carbapenem resistance, and the detection and monitoring of CRGs at the environmental and farm levels could aid in preventing the spread of resistance to consumers [59].

Key environmental changes could impact the spread and prevalence of AMR, including climate warming, the loss of biodiversity, and the emission of pollutants into the environment [60]. As global temperatures rise, it is hypothesized that AMR also increases in humans, animals, and the environment due to the facilitation of bacterial growth in environmental reservoirs and increased horizontal gene transfer [60,61]. A study conducted using data from the China Antimicrobial Surveillance Network and annual average ambient temperatures found that a 1 °C rise in the regional temperature was associated with a 1.14-fold increase in the CR *K. pneumoniae* prevalence and a 1.06-fold increase in the CR *Pseudomonas aeruginosa* (*P. aeruginosa*) prevalence from 2005 to 2019. [62]. Another study found that from 2000 to 2016, across 28 European countries and in about four million bacterial isolates, there was a 0.33% to 1.2% elevation per year in the resistance to aminoglycosides, cephalosporins, and fluroquinolones in *Escherichia coli* (*E. coli*) and *K. pneumoniae*, with a 10 °C rise in temperature [63]. In the U.S., a study found that the spread of AMR can potentially be accelerated by warming temperatures and may elevate resistance to some antibiotics by up to 10% [64]. The study used 1.6 million bacterial isolates *of E. coli*, *Klebsiella*, and *Staphylococcus aureus* and found that a 10 °C rise in temperature from 1980 to 2010 could be associated with an increase in antibiotic resistance of 5.1%, 3.4%, and 3.1%, respectively. In addition to the impacts of elevated temperatures, antibiotic residues in the environment also create selective pressure on bacteria, which could worsen resistance [65]. Increased antibiotic consumption causes more antibiotic waste and environmental contamination [66]. The growing frequency of severe weather conditions caused by global warming, like floods, could also accelerate the spread of resistant bacteria, especially soil-derived bacteria [67]. It was shown that untreated sewage overflow after stormwater events is a main source of the increased spread of antibiotic resistance genes to nearby rivers [68]. Studying the effects of climate change on the spread of AMR is important in determining preventative measures that will decrease AMR overall in the future.

### 2.3. Surveillance and Prevalence of CR

The first global AMR standardized surveillance effort was established by the WHO in 2015, when the Global Action Plan (GAP-AMR) and the Global Antimicrobial Resistance and Use Surveillance System (GLASS) were approved [69]. The goal of the GLASS is to monitor AMR in humans, the food chain, and the environment and provide standardization for AMR data collection to improve existing surveillance efforts, especially in low-income countries [69,70]. Before this standardized program was established, in 2004 Pfizer created the Antimicrobial Testing Leadership and Surveillance (ATLAS) program, that monitors clinical isolates for AMR [71]. Currently, in the U.S. changes in foodborne antimicrobial susceptibility are tracked by the National Antimicrobial Resistance Monitoring System (NARMS) that collaborates with the U.S. Food and Drug Administration (FDA), the Centers for Disease Control and Prevention (CDC), and the U.S. Department of Agriculture (USDA) [72,73,74]. The CDC’s component of the NARMS conducts susceptibility testing on human isolates, while the FDA conducts serotyping, antimicrobial susceptibility testing, and genetic analysis on retail meat samples, and the USDAconducts testing from food-producing animal samples [73]. The WHO determined that new antibiotics are critically needed for CR *Acinetobacter baumannii* (CRAB), *P. aeruginosa* (CRPA), and *Enterobacteriaceae* (CRE) due to rising levels of AMR in these pathogenic bacteria [75].

The prevalence of CR bacteria varies depending on country or region, infection type, bacteria, and antibiotic according to the 2021 GLASS AMR global summaries data [76]. This report provides an estimated box plot of the proportions of patients with resistant infections caused by different bacterial species; however, these data have not been verified and require further investigation. Table 1 details the estimated median percentage of patients with carbapenem-resistant infections caused by *Acinetobacter* spp., *E. coli*, and *K. pneumoniae* in blood samples, based on reported global data.

*Acinetobacter* spp. are a concerning group of bacteria due to the high prevalence of carbapenem resistance. One study, using data from the ATLAS program and the five global regions of Asia/Pacific (APAC), Europe (EUR), Latin America (LATAM), Middle East/Africa (MEA), and North America (NA), found that the percentage of CRAB isolates remained relatively constant per region [77]. The estimated percentages of CRAB isolates in 2022 were approximately 27% in NA, 52% in EUR, 53% in APAC, 64% in MEA, and 68% in LATAM [77]. A total of 13,500 isolates from medical centers across 49 countries were included as part of this dataset [77].

Another concerning group of bacteria is CRE. *Enterobacteriaceae* include Gram-negative bacilli like *E. coli*, *K. pneumoniae*, *E. cloacae*, and *Citrobacter* spp. [78]. *K. pneumoniae* are the most common *Enterobacteriaceae* containing CR resistance genes [79]. One study shows that in the U.S. there is an endemic distribution of KPC-producing CRE and sporadic occurrences of IMP-, NDM-, OXA-, and VIM-producing CRE [79]. Another study showed that in the U.S., in 2020, the overall crude, or total number of cases, CRE incident rate per 100,000 population was 6.08 [80]. This study obtained data from seven different testing sites from Colorado, Georgia, Tennessee, Maryland, Minnesota, New York, and Oregon that conduct CRE monitoring through the CDC’s Emerging Infections Program (EIP) [80]. Another study using global data from the ATLAS program found that CRE isolates increased in LATAM, APAC, EUR, and MEA and decreased in NA from 2018 to 2022 [77]. The estimated percentages of CRE isolates in 2022 were approximately 1% in NA, 4% in EUR, 11% in MEA and APAC, and 13% in LATAM [77].

**Figure 2:**
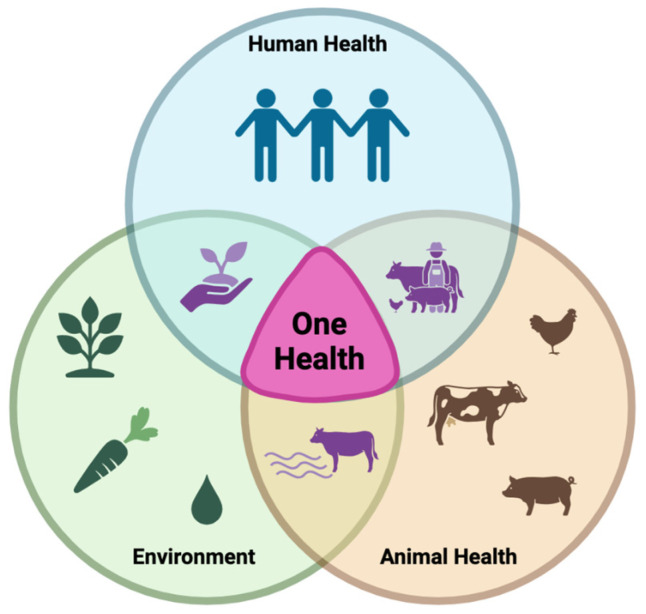
The One Health strategy for solving global challenges is based on the interconnectedness of human health, animal health, and the environment and promotes collaboration across all disciplines (original image created with https://www.biorender.com/, URL accessed on 12 June 2025).

The spread and prevalence of CR bacteria can be influenced by hospital and environmental conditions such as overcrowding, inadequate cleanliness, and poor infection control practices [81]. Socioeconomic factors, such as poor sanitation, a limited access to clean water, uncontrolled antibiotic use, and the cohabitation of humans and animals, can contribute to regional differences in the CR prevalence [82]. One Health is an integrated approach that brings together multiple sectors and disciplines at local, regional, national, and global levels [83]. According to the CDC, there is no single inventor of One Health; however, in 1964, Dr. Calvin Schwabe coined the term “One Medicine” and called for a collaborative effort in studying diseases that affected animals and humans [84]. Its overall goal is to achieve optimal health outcomes by acknowledging the linkage between people, animals, plants, and the environment [83]. This concept is illustrated in Figure 2.

The One Health approach applies to AMR because it is a complex issue that affects human, animal, and environmental health [85]. The majority of antimicrobial classes are used in animals and humans, and some are also used in horticulture for plant disease prevention [86]. One Health approaches are essential for addressing AMR by analyzing how human activities and antibiotic use influence surrounding environments, contributing to AMR emergence and transmission [87,88]. A One Health surveillance approach would need to include standardized AMR data and a shared data system across all three sectors to potentially track AMR as it moves through each sector and further understand key drivers and risk factors for its transmission [89]. This collaborative approach would strengthen AMR surveillance and provide a deeper understanding of the success rates of mitigation strategies and the potential direction for future the One Health paradigm.

## 3. Current Standardized Methods of AMR Detection in Bacteria

Current standardized detection methods for AMR can be divided into phenotypic and genotypic tests [90]. Phenotypic or culture-based AST methodologies are commonly adapted from the Clinical and Laboratory Standards Institute (CLSI) and the European Committee for Antimicrobial Susceptibility Testing (EUCAST) [91]. In genotypic testing, the genes that code for bacterial resistance enzymes are detected [92]. These methods include polymerase chain reaction (PCR)-based methods, whole-genome sequencing (WGS), targeted sequencing, and metagenomic sequencing [93]. Traditional standardized methods for AMR testing refer to laboratory procedures that have been widely accepted and validated by organizations like the CLSI and EUCAST.

### 3.1. Phenotypic Detection Methods

The goal of AST is to determine whether bacterial growth is affected in the presence of antimicrobials and include dilution-based and diffusion-based tests [93]. Results from these methods are interpreted according to clinical breakpoints provided by standards organizations such as the CLSI and EUCAST, which define minimum inhibitory concentrations (MICs) or inhibition zone diameters used to classify bacterial susceptibility [93]. The EUCAST methodology is preferred in Europe, and the CLSI is primarily used in the U.S. and other non-European countries [91]. Current phenotypic susceptibility testing requires pure culture bacterial growth after biological isolation from clinical samples, which can take 24 to 48 h [92]. This involves enrichment and isolation steps, including a blood culture or overnight culture on selective agar plates, in addition to antibiotic susceptibility testing (AST) [90]. Conventional methods of AST include disk diffusion, broth macrodilution and microdilution, and gradient diffusion, and the procedures, including culturing and isolation, can take a minimum of 1 to 3 days [90].

Disk diffusion, used by both the CLSI and EUCAST, is one method of phenotypic AST, and it works for a wide range of bacteria and does not require special equipment [94]. Overnight growth of a 16–24 h incubation on a non-selective medium is used to prepare a culture in sterile saline or a buffer, and the suspension is spread on the surface of an agar plate [95]. Antibiotic disks are put on the agar surface, and the plates are incubated overnight [95]. After the incubation, inhibition zone diameters are measured from the center of the antibiotic disk and are then interpreted as susceptible, intermediate, or resistant according to CLSI or EUCAST clinical breakpoints [95]. These breakpoints are unique for each bacterium–antibiotic combination and also depend on the source of the clinical infection [96].

Another method of phenotypic AST is broth microdilution. An overnight bacterial culture is prepared and diluted to around 10^5^ CFU/mL and placed into 96-well plates containing different concentrations of antimicrobials, so that each well has a different antibiotic concentration [97]. The plates are allowed to grow overnight, and the MIC is measured for individual wells to determine the susceptibility or resistance of the bacteria [97].

Another phenotypic AST dilution-based method is broth macrodilution. Tubes containing serial dilutions of antimicrobial agents in broth are inoculated with a known quantity of suspended bacteria, typically around 10^5^ CFU/mL [98]. The bacterial growth can be measured by the turbidity of the solution, after 24 h, and provides MIC values [98]. The turbidity of suspended microorganisms can be visually compared to McFarland standards to estimate the concentration of the suspended bacteria [99].

The Epsilometer test (E-test) utilizes plastic strips that have an antibiotic agent gradient on one side, which is released onto the agar with contact [100]. Bacterial cultures are spread onto agar plates, and an E-test strip is put on the surface [101]. The plates are incubated for 24 h, and then MICs are read from the test strip where the zone of inhibition intersects the MIC scale on the strip [101]. This method is similar to disk diffusion but provides a quantitative result for the MIC instead of a qualitative result of susceptible, intermediate, or resistant [100].

An agar dilution is another method of phenotypic AST. Different concentrations of antimicrobial substances are combined with liquid agar solutions, which are dispensed into individual agar plates [97]. Bacterial samples are diluted to obtain isolated colonies, and the plates containing the antibiotic and control plates not containing antibiotics are inoculated and incubated overnight [97]. After the incubation, the bacterial growth is determined by the number of colonies on the plates and is compared between plates containing antimicrobial agents and control plates [97].

### 3.2. Genotypic Detection Methods

The PCR is a nucleic acid amplification technique used to detect components of deoxyribonucleic acid (DNA) or ribonucleic acid (RNA) [102]. The DNA polymerase I enzyme, or Taq DNA polymerase, is used to denature and renature segments of DNA or RNA to allow for amplification and detection [102]. The three phases of the PCR include the denaturation of the template DNA into single strands, the annealing of primers to the original strand, and the elongation of the DNA strands from the primers [103]. During denaturation, the DNA is heated to around 95 °C, which breaks the hydrogen bonds between base pairs of the double-stranded DNA [102]. Then, specific primers bind to the single-stranded DNA at complementary sites during annealing, at temperatures around 55 and 72 °C [102]. Then DNA polymerase synthesizes new DNA strands identical to the original strand in the amplification step [102]. This process is repeated for multiple cycles, using a thermal cycling device [102]. After amplification occurs, PCR products can be analyzed using gel electrophoresis [103]. The advantage over phenotypic detection methods is the ability to amplify the genes from organisms that are not viable or dead, which decreases the chances of receiving a false negative result based solely on phenotypic testing and characteristics [104]. The real-time PCR, also called a quantitative PCR (qPCR), is an alternative option and can detect target DNA segments more rapidly than the traditional PCR and gel electrophoresis [102]. The real-time PCR utilizes fluorogenic labels to quantify the amount of amplified target DNA in the sample [105]. The real-time PCR is more expensive than conventional PCRs and requires specific instruments to complete; however, it provides a quantitative measure that the conventional PCR does not [102]. WGS uses programs based on a De Bruijn graph (DBG), and resistant genes can be found by annotating sequences [104]. These searches are performed by comparing sample genes to known genes in reference databases and annotating similarities [104]. WGS uses short-read sequencing of DNA, which is assembled into contiguous sequences or contigs representing the genome [106]. However, when there are repetitive or complex regions, these contigs are often fragmented, and it is difficult to tell whether it came from a chromosome or plasmid [106,107]. WGS produces the complete genetic makeup of an organism using automated computer systems; however, it can be slow, labor-intensive, and costly [108].

### 3.3. Limitations of Current Methods

Because traditional phenotypic AST methods, such as disk diffusion and broth dilution, require bacterial cultivation, the time required to complete the tests is a significant downside [104]. Other disadvantages include the inability to detect non-cultivable bacteria, the labor-intensive steps required for testing, and the high consumption of laboratory materials [104,109]. The reliability of genotypic AST methods such as the PCR depends on several factors, including the quality of raw material samples, cycling conditions, temperature settings, primer concentrations, and DNA extraction procedures [104]. The PCR is also not always an accurate measure of susceptibility, because the absence of a resistance gene does not guarantee the organism is susceptible to an antibiotic, because there are many different resistance mechanisms [110]. On the other hand, the gene presence does not guarantee gene expression in an organism, so the bacterial response to an antibiotic may not always align with gene presence [111]. It was found that for Gram-positive bacteria, a single mechanism of resistance causes the most clinically significant resistance, which resulted in a 98 to 100% accuracy for predicting the phenotypic susceptibility from genotypes [112]. However, predictions in Gram-negative bacteria may have a lower accuracy due to more complex resistance mechanisms [112]. For example, one study found that 72–76% of *E. coli* that was predicted to be resistant was found to be phenotypically resistant [113]. WGS is an expensive, time-consuming technique compared to other traditional methods of AST [109]. It also requires a large data storing capacity and extensive data analysis and often results in fragmented sequences of an unknown origin [106,107,114]. The estimated time required for each testing method is listed in Table 2.

Another challenge in clinical microbiology is identifying antibiotic resistance in populations that are heteroresistant to specific antibiotics. This means that in one bacterial population, there could be subpopulations that have differing responses and susceptibility to antibiotics, which can cause difficulties in accurately describing a bacterial resistance profile and treatment failure [117]. Clinical susceptibility tests often start with 10^6^ CFU/mL, and if a resistant subpopulation exists at a frequency of only 10^−6^ CFU/mL, there is only one out of a million cells that has resistance to that antibiotic, and it is unlikely to be detected [118]. In immunocompromised patients, AMR is a higher risk, due to the decreased immune system responses and potential of prolonged periods of infection [119]. This is also true for young children (<5 years), older adults (>65), and people in low-income areas [120]. Heteroresistant bacterial populations are particularly concerning for immunocompromised patients, because the resistant subpopulations can expand during or after antibiotic exposure, potentially resulting in treatment failure [121]. Heteroresistance was associated with an increased risk of poor clinical outcomes in immunocompromised patients, who had a poor initial treatment response and infection recurrence after treatment [118]. Studies have shown that heteroresistance can be frequent among clinical samples. One study found that 27.5% of 138 *E. cloacae* complex isolates from clinical samples had phenotypic heteroresistance [122]. Another study found that the heteroresistance to colistin was more common (10.1%) than conventional homogenous resistance (7.1%) in 408 carbapenem-resistant *Enterobacterales* isolates in the U.S. [123]. The development of more sensitive methods to detect resistance and heteroresistance is essential for improving treatment outcomes.

## 4. Emerging Technologies for CR Detection in Bacteria

The use of machine learning (ML) has increased in the field of disease diagnosis in healthcare, due to the costly, time-consuming, and error-prone nature of traditional diagnostic techniques [124,125]. ML is an area of artificial intelligence (AI) that uses data inputs to generate predictions using algorithms and statistical models without the need for specific instructions, therefore independently improving its performance [126]. These algorithms can detect patterns in antibiotic usage and resistance to predict which microorganisms are likely to develop resistance to specific antibiotics [127]. They can also predict protein functions and gene expression [126]. One study aimed to predict CR at the time of culture collection, using a ML model, and determined that certain cohorts of patients with infections were more likely to develop CR [128]. During the study of patients with existing CR infections, they were more likely to be male, have had a previous hospital stay in the last 180 days, have had taken antibiotics in the last 180 days, have had a central venous catheter, and had surgery [128]. The most common sources of CR were *Pseudomonas* spp., *Enterobacter* spp., and *Acinetobacter* spp. from respiratory and urinary tracts [128]. Another study utilized the Generative Pre-trained Transformer (GPT)-4 to interpret results from the EUCAST disk diffusion testing to estimate the likelihood of the presence of beta-lactamases in Gram-negative bacteria [129]. The AI system analyzed images of the disk diffusion images to create a table of likelihoods for the bacteria having extended-spectrum beta-lactamase, plasmid-mediated AmpC, and carbapenemase production [129]. This study compared the human analysis to the AI system and resulted in a 95.5% and 100% sensitivity and a 98.5% and 98.8% specificity for the human analysis and AI analysis, respectively, for the detection of carbapenemases [129]. Although these ML and AI strategies can improve diagnostics and antimicrobial susceptibility testing, they require expensive infrastructure, large genomic datasets, and knowledgeable personnel to function properly [127,128,130].

Reporter phage-based diagnostics show potential in rapid susceptibility testing and pathogen detection [131]. Phages are viruses that use host bacterial cells for propagation [132]. They bind to bacterial surfaces using receptor binding proteins, inject their DNA or RNA into the host cell, then utilize the cell’s functions to propagate their genetic material [133]. Phages can be genetically engineered to carry reporter genes that are replicated and expressed when the phage infects a bacteria, which produces a detectable signal [133,134]. They have efficient mechanisms for attaching to a target bacteria with high specificity, even in complex matrices [135]. Phages have been used in several bacterial identification assays [135,136], but the detection of carbapenem and antibiotic resistance in bacteria is a less studied field. One study demonstrated the successful rapid identification of carbapenem-resistant *K. pneumoniae* utilizing a reporter phage detection method [131]. In this study, the researchers used a genetically engineered phage to infect *K. pneumoniae* K64 and produce luminescence if the host bacteria were alive and allowed the phage to replicate [131]. The resistance profile was estimated based on the level of luminescence that occurred in the presence of various antibiotics, indicating the level of surviving bacteria [131]. The researchers detected *K. pneumoniae* at a low concentration of one CFU per well when using pure cultures and determined through the luminescence analysis that the specific strain was resistant to levofloxacin, ceftazidime, streptomycin, tigecycline, ciprofloxacin, trimethoprim, and ertapenem [131]. Another study developed a phage-based AST assay to determine the resistance profiles and MICs of *Yersinia pestis* to multiple antibiotics and successfully detected 59 strains with a detection limit of detection of 1 × 10^6^ CFU/mL, directly from clinical and environmental samples [137]

Raman spectroscopy is an analytical technique used for the study of the molecular composition and structure of samples by observing how light interacts with them [138]. A bacterial cell’s DNA, proteins, lipids, and other biomolecules contribute to its Raman spectrum and creates a unique molecular fingerprint that can be used for identification [138]. A spectral database that links antibiotic treatments and mechanisms of action to specific bacterial species could enable the rapid identification of bacteria and their antibiotic susceptibility profiles [138]. One study utilized surface-enhanced Raman spectra (SERSs) coupled with an ML data analysis to distinguish carbapenem-resistant and carbapenem-susceptible *K. pneumoniae* [139]. The researchers found that there were eight ML methods that could successfully predict the carbapenem susceptibility and resistance in *K. pneumoniae*, based on SERS spectral data, that resulted in prediction accuracies ranging from 93.54% to 100% from bacteria isolated from clinical samples [139]. Another study using SERSs identified CRE and their carbapenem-resistant enzymes, KPC and NDM. In this study, *Enterobacter aerogenes* (*E. aerogenes*) and *K. pneumoniae* strains were analyzed and it was found that strains expressing the same enzyme showed a minimal spectral variation and strains expressing different enzymes showed some spectral differences [140]. Machine learning was integrated with this SERS analysis and was able to identify CRE based on spectral profiles, and resulted in a classification accuracy for different CRE strains and their enzyme-producing subtypes of 94% and 96.13% in pure cultures [140]. Another study utilized SERSs with gold nano-stars to detect carbapenemase activity in CRE by observing the change in molecular properties of bacterial cells when exposed to carbapenem antibiotics [141]. Gold nano-stars were used to amplify the Raman signal, due to their morphology of multiple branches’ sharp tips [141]. The researchers combined an antibiotic with these gold nano-stars to allow for the detection of enzymatic hydrolysis using the SERS. This allowed for the differentiation between *E. coli* containing NDM and not containing NDM [141]. There are very few studies claiming to detect resistant subpopulations in heteroresistant bacteria; however, one study utilized a Raman-based antibiotic susceptibility test successfully, producing results in 3.5 h [142]. The researchers evaluated the bacterial metabolic activity using deuterium labeling and Raman spectroscopy [142]. This study was able to detect heteroresistant bacteria at a frequency as low as 10^−6^ resistant bacteria in clinical isolates [142].

Clustered regularly interspaced short palindromic repeats (CRISPR) systems use RNA to guide Cas enzymes to bind and cleave sections of DNA or RNA sequences and can be used for genome editing, gene expression regulation, and nucleic acid detection [143]. CRISPR-based DNA detection methods utilize fluorescent reporters to indicate when a specific target sequence has been located [143]. The CRISPR system has the potential for single-nucleotide specificity, which can be utilized for antibiotic resistance detection [143]. CRISPR-based diagnostics have recently been used for carbapenem resistance detection. One study detected OXA-48 and GES carbapenemases in *K. pneumoniae*, *P. aeruginosa*, *E. cloacae*, and *Serratia marcescens* using a CRISPR-Cas13a-based assay in clinical samples with 100% specificity and sensitivity [144]. Another study utilized CRISPR-Cas13 paired with a PCR and recombinase-aided amplification (RAA) to detect KPC and NDM in carbapenem-resistant *K. pneumoniae* with a sensitivity and specificity of 100% [145]. Another study detected *bla*_NDM_ in CRE in food samples using a PCR-CRISPR-Cas12a-based assay [146]. The study showed that this system produced a higher sensitivity and specificity compared to the conventional PCR and gel electrophoresis [146].

## 5. Application of Biosensors for Pathogen and AMR Detection

Biosensors are analytical tools that can detect biological components and are used in disease monitoring, the detection of pollutants and pathogens [147]. They typically contain a target analyte, a bioreceptor involved in a bio-recognition interaction, a transducer, and a signal generation component [147]. Important characteristics of biosensors include selectivity, reproducibility, stability, and sensitivity [147]. Nanoparticles can be used as transducing materials in biosensor applications and can be composed of metal, metal oxides, carbon, or organic matter and have distinctive magnetic, electric, and optical properties [148]. Due to these properties, the large area-to-volume ratios, and their reactivity, nanoparticles have great potential for infectious disease diagnostics and biosensing abilities [148]. The type of transducing material categorizes biosensors and includes electrochemical, optical, and mass-based biosensors [149]. Electrochemical biosensors convert biological responses into electronic signals and produce rapid results, have simple procedures, and are low-cost and portable [149,150]. Optical biosensors generate visible results, like color changes or fluorescence, and produce rapid results and sensitive measurements, while being low-cost and portable diagnostic tests [151,152]. Mass-based biosensors, like piezoelectric biosensors, have simple procedures and are low-cost [153]. Several examples of optical, mass-based, and electrochemical biosensors used for diagnostic testing are listed in Table 3.

### 5.1. Biosensors for AMR Detection

Biosensors have the potential to be helpful diagnostic tools due to their simple and portable methods, inexpensive materials, rapid results, and high sensitivity [162]. To be portable, these systems must function without the reliance on bulky instrumentation [163]. For example, instead of requiring a benchtop spectrophotometer, a portable optical biosensor could leverage a smartphone camera and app to detect and quantify colorimetric changes in the sensor output. There have been several recent advancements in AMR detection in bacteria, using biosensors. One example is a dual-channel electrochemical biosensor simultaneously targeting the EspB antibody to identify the virulence of enteropathogenic *E. coli* and the beta-lactamase substrate to identify resistance in less than three hours [164]. In the presence of beta-lactamase-producing bacteria, nitrocefin is hydrolyzed, leading to a visible color change. By comparing the colorimetric shift between hydrolyzed and non-hydrolyzed nitrocefin, the biosensor can determine the beta-lactamase activity and indicate resistance. The assay was performed using pure cultures and achieved a detection sensitivity of 3.6 ng/mL [164]. Another electrochemical biosensor evaluated bacterial activity in the presence of antibiotics to determine the resistance profile [165]. This biosensor utilized graphene dispersion to coat *E. coli* cells from pure cultures to enhance the signal of the electrocatalytic reduction current and determine the level of bacterial activity in the presence of antibiotics [165]. The biosensor measured changes in this current as an indicator of the metabolic activity of the bacteria, and when they were exposed to effective antibiotics, their metabolic activity and the reduction current decreased. This testing was performed in pure cultures and required 1 × 10^7^ CFU of bacteria [165]. One example of an optical biosensor for AMR detection involves a multi-channel surface plasmon resonance (SPR) platform for the detection of methicillin-resistant and -susceptible *S. aureus* and vancomycin-resistant and -susceptible *Enterococcus* [166]. This study examined SPR angles through the channels during an exposure to the antibiotic and was able to distinguish resistant from susceptible bacteria by characterizing the change in SPR angles with bacterial responses to the antibiotic [166]. This test was completed in three hours for the pure culture *S. aureus* and six hours for the *Enterococcus* resistant and susceptible differentiation [166].

Biosensing technology has only recently been developed for specifically detecting carbapenem resistance. One example is a thermometric biosensor that detects the amount of beta-lactam antibiotic remaining in a system after *bla*_NDM-1_-mediated hydrolysis [167]. The biosensor measures the heat of the enzymatic reaction present as the antibiotic is broken down and signals how much antibiotic is left in a sample, indicating the enzyme activity and beta-lactam resistance profile [167]. This test was able to be analyzed in less than one hour using an assay algorithm and had a 100% accuracy when tested with clinical isolates [167]. Other studies utilized a modified GoldNano Carb test to estimate the acid production from carbapenem hydrolysis using GNPs as a signal for pH levels [168,169]. Each test was less than USD 0.25 and was able to successfully detect carbapenemase-producing *Enterobacteriaceae* clinical isolates, resulting in a sensitivity of 99% [169]. Another example uses a chemical sensor called an Ion Sensitive Field Effect Transistor (ISFET) to detect carbapenem hydrolysis [170]. This ISFET sensor was able to detect multiple beta-lactamase enzymes, including NDM-1, NMC-A, IMP-1, KPC-2, OXA-48, and VIM-1, at varying minimal concentrations [170]. This method resulted in 100% sensitivity for NDM, IMP, and KPC and 77.3% for VIM producers, while the OXA producers failed to be detected in clinical isolates [170].

### 5.2. Gold Nanoparticles and Gold Nanoparticle-Based Biosensors

Nanoparticle-based biosensors with colorimetric properties create visible signals, and gold nanoparticles (GNPs) are commonly used in biomedical applications, like diagnostic testing, due to their distinctive surface plasmon resonance (SPR), strong absorption in visible light, and biocompatibility [99,171]. GNPs have been used in antibiotic research and are reported to be efficient drug delivery vehicles [172]. Antibiotics can adhere to and modify the surface of GNPs, which leads to an improved drug bioavailability and penetration through bacterial cell walls, therefore increasing the antibacterial activity [165]. GNPs have also been used in biosensor applications for detecting target nucleic acid in paper-based assays combined with a loop-mediated amplification (LAMP), where the biosensor aids in the DNA or RNA extraction, amplification, and detection [148,172,173]. Another method utilizing GNPs to create an optical result involves biotin-labeled amplicons with the GNP detection probes immobilized in the test zone, and a visible signal is generated for a target bacterium [174]. Colorimetric biosensors have simple procedures and are low-cost, and results can be visually and easily interpreted [175]. For example, plasmonic, GNP-based biosensors have been used to successfully detect the *uid*A gene in *E. coli*, differentiating samples with *E. coli* from those containing *Salmonella* Enteritidis, *K. pneumoniae*, and *E. cloacae* in pure cultures and bacteria extracted from food samples [176]. This study resulted in a sensitivity of 93.3% and a specificity of 99% when using clinical isolates [176]. A similar assay was also designed to detect pathogenic *E. coli* O157:H7 by targeting the *stx1A1* gene and distinguished *E. coli* O157:H7-containing samples from those containing *Listeria monocytogenes* and non-pathogenic *E. coli* C-3000 [161]. This study resulted in a limit of detection of 2.5 ng/μL [161]. GNP-based biosensors have also been used to detect carbapenem resistance, specifically the *bla*_KPC_ gene, from multiple isolated bacterial cultures and species [177]. One study designed a GNP-based lateral flow biosensor combined with multiple displacement amplification to simultaneously detect *A. baumanii* and the presence of *bla*_OXA-23-like_ genes [178]. This resulted in a 100% specificity when using bacterial isolates and a limit of detection of 100 fg of genomic templates per reaction [178]. This GNP-based biosensor technology can be applied to the detection of CR genes resulting in a portable, rapid, and inexpensive detection method that can be utilized in environmental and agricultural settings.

## 6. Discussion

There are several phenotypic, genotypic, and emerging technologies related to the detection of AMR and carbapenem resistance. Each method has unique advantages and disadvantages, and these are summarized in Table 4.

The strengths of phenotypic methods are that they are well established with widely accepted standards and regulations and are relevant in clinical settings. Due to the time and labor required for phenotypic testing, it may not be the optimal testing method for rapid and portable applications in AMR monitoring and diagnosis. Genotypic testing provides rapid results that do not require bacterial cultivation. These methods are useful for AMR surveillance and gene discovery; however, they may be less useful for treatment guidance due to the potential inconsistency of the genotype and phenotypic expression. Emerging methods of AMR detection demonstrate potential for predicting resistance quickly and avoiding bacterial cultivation. One advantage of developing biosensing techniques for diagnostic assays is the versatility of the applications of biosensors [188]. Biosensors can be designed to be low-cost, portable, highly sensitive, user-friendly, and capable of multiplex detection [188,189]. The further development of biosensors for AMR detection, and specifically carbapenem resistance, will be beneficial in certain applications due to their unique properties and advantages over traditional AMR detection methods. Additional verification is needed for biosensing applications and emerging technologies related to AMR detection in real samples, with heteroresistant bacterial samples, clinical validation, and in resource-limited settings.

## 7. Conclusions

AMR is a rapidly growing global issue and affects millions of people every year. The increased use of antibiotics in agricultural and clinical settings and the lack of surveillance of AMR are causing the emergence of highly resistant human pathogens. The increased resistance to carbapenems, a subclass of antibiotics, is a significant concern due to their clinical relevance as a reliable last-resort antibiotic for serious infections. Carbapenem-resistant organisms have been detected in the environment and pose a significant threat to human health, primarily due to the dissemination of resistance genes and bacteria that can cause human infections. The rapid detection of carbapenem-resistant bacteria is essential for making informed diagnostic decisions about medications and treatment approaches for patients with life-threatening infections and for AMR surveillance initiatives. A variety of methods exist for AMR monitoring and diagnosis, each with unique strengths and limitations. Although existing methods for detecting AMR genes, like PCR-based methods, are more accurate and faster than traditional culture-based phenotypic AST, they remain relatively expensive and time-consuming, and they do not necessarily confirm phenotypic resistance. Emerging technologies including methods utilizing machine learning, CRISPR, Raman spectroscopy, and biosensing techniques show promise for AMR detection, especially in low-resources settings, due to their speed and portability.

## Figures and Tables

**Table 1:** The estimated global median percentage of patients with infections resistant to meropenem, imipenem, doripenem, and ertapenem caused by different pathogens in blood samples and the number of countries that submitted data to this report (*n*).

Pathogen	Meropenem	Imipenem	Doripenem	Ertapenem
*Acinetobacter* spp.	64.29% (*n* = 57)	64% (*n* = 55)	54.67% (*n* = 4)	Not reported
*E. coli*	0.5% (*n* = 58)	0.5% (*n* = 60)	8.2% (*n* = 4)	1.2% (*n* = 45)
*K. pneumoniae*	11% (*n* = 59)	8.8% (*n* = 58)	14.5% (*n* = 5)	17% (*n* = 45)

**Table 2:** Traditional standardized methods of AST and the time it takes to obtain results from bacterial pure cultures.

Method	Time to Result	Source
Phenotypic		
Disk Diffusion	16–20 h	[94]
Broth Microdilution	24–48 h	[97]
Broth Macrodilution	24 h	[98]
Epsilometer Testing	24 h	[101]
Agar Dilution	12–24 h	[97]
Genotypic		
PCR-Based Methods	4–8 h	[115]
WGS	1–6 d	[116]

**Table 3:** Examples of diagnostic biosensors utilized for pathogen detection.

Type of Biosensor	Transduction	Target	Source
Porous silicon (PSi)-based biosensor	Optical	Detection of *E. coli*, *S. aureus*, *K. aerogenes*, and *B. subtilis*	[154]
DNA-based piezoelectric biosensor	Mass-based	Simultaneous detection and genotyping of Human Papilloma Virus (HPV)	[155]
Nanoparticle-based lateral flow biosensor	Optical	Detection of target genes in HIV-1	[156]
Silver nanoparticle-based surface plasmon resonance (SPR) biosensor	Optical	Detection of *E. coli* by enhancing SPR signals with L-His-capped silver nanoparticles	[157]
Electrochemical CRISPR biosensor	Electrochemical	Detection of target genes in methicillin-resistant *S. aureus* (MRSA)	[158]
DNA-based plasmonic biosensor	Optical	Detection of thermonuclease (nuc) gene in *S. aureus*, invA gene in *Salmonella*, and StxA1 gene in *E. coli* O157	[159,160,161]

**Table 4:** Advantages and disadvantages of current phenotypic AST, genotypic methods, emerging detection methods, and biosensors for detecting antibiotic resistance.

AMR Detection Method	Advantages	Disadvantages	Sources
Phenotypic Methods			
Disk diffusion	Inexpensive, allows visibility of growth, and direct susceptibility testing, clinical relevance for bacterial response to antibiotic	Time-consuming, not standardized, qualitative results, requires cultivable organisms	[109,179]
Broth dilution	Standardized, accurate results, quantitative results, clinical relevance for bacterial response to antibiotic	Time-consuming, complicated procedure, laborious, requires cultivable organisms	[180,181]
Agar dilution	Standardized, high reproducibility, quantitative results, clinical relevance for bacterial response to antibiotic	Time-consuming, complex procedure, laborious, requires cultivable organisms	[109,180,181]
Epsilometer test	Simple procedure, flexible applications, quantitative result, clinical relevance for bacterial response to antibiotic	Time-consuming, moderately expensive, requires cultivable organisms	[182,183]
Genotypic Methods			
PCR-based detection	Rapid, high specificity, ability to detect AMR in non-cultivable organisms	Complicated procedure, laborious validation, sensitive to experimental conditions, expensive, genotypic result does not guarantee phenotypic response	[104,111]
Whole-genome sequencing	High accuracy, sensitive, ability to detect non-cultivable organisms	Time-consuming, expensive, requires known resistance genes, complex data analysis, qualitative results, genotypic result does not guarantee phenotypic response, fragmented genomic sequences	[106,107,109,111]
Emerging Methods			
Machine learning and artificial intelligence-based assays	Rapid, predictive, and real-time results	Requires existing large databases and ML algorithms, data privacy concerns	[125,128]
Phage-based assays	Rapid, sensitive, inexpensive, accurate	Requires cultivable organisms, requires high bacterial concentration	[134]
Raman spectroscopy-based assays	Rapid, real-time results, specific, accurate, and shown to successfully detect resistance in subpopulations of heteroresistant bacteria	Potentially damages samples with laser exposure, requires cultivable organisms, requires large databases and machine learning algorithms	[142,184]
Clustered regularly interspaced short palindromic repeats-based assays	Inexpensive, do not require special equipment, simple procedures	Can be unstable, require complicated sample processing, often require pre-amplification	[185]
Biosensors			
Dual-channel electrochemical biosensor	Low detection limit, portable, detects virulence and antibiotic resistance markers simultaneously	Time-consuming, complicated procedure, non-specific binding, requires cultivable organisms	[164]
Gold nanoparticle-based lateral flow biosensor	Rapid, identifies two targets simultaneously, simple procedure	Qualitative or semi-quantitative result, complex device structure, can be affected by sample matrix	[178,186]
Surface plasmon resonance multi-channel sensor platform	Rapid, simultaneous detection capability, less laborious procedure than similar existing methods	Requires special equipment, qualitative or semi-quantitative results, abnormalities in bacterial adhesion can affect results	[166]
Gold nanoparticle-based optical biosensors	Rapid, inexpensive, simple procedures, accurate	Lack of testing in real-world applications	[177,187]

## Data Availability

No new data were created or analyzed in this study. Data sharing is not applicable to this article.
